# Dilemmas and limitations interpreting carbohydrate antigen 19-9 elevation after curative pancreatic surgery: A case report

**DOI:** 10.1016/j.ijscr.2018.11.022

**Published:** 2018-11-20

**Authors:** Grace Gold, Su Kah Goh, Christopher Christophi, Vijayaragavan Muralidharan

**Affiliations:** a Hepato-Pancreato-Biliary & Transplant Unit, Austin Hospital, Melbourne, Australia; b The University of Melbourne, Department of Surgery, Austin Hospital, Melbourne, Australia

**Keywords:** Pancreatic cancer, CA19-9, Tumour marker, General surgery, Hepatopancreatobiliary surgery, Case report

## Abstract

Introduction: Serum carbohydrate antigen 19-9 (CA19-9) is routinely used in the management of pancreatic cancer for diagnosis, prognostication and surveillance purposes despite its low specificity.

Case presentation: This case report demonstrates the challenges of interpreting elevated serum CA19.9 levels in a patient with pancreatic cancer. We performed a Whipple’s procedure in an otherwise fit 78-year-old man for pancreatic adenocarcinoma. One year after surgery, he presented with a non-malignant anastomotic stricture that was associated with an elevated serum CA19-9. Subsequently, he presented with biliary sepsis secondary to an infected liver cyst. CA19-9 in the cyst fluid and serum were elevated. On both of these occasions, serum CA19-9 normalised without any clinical or radiological evidence of recurrence.

Discussion: Despite sound clinical acumen and investigations, it may be hard to differentiate cancer recurrence from benign aetiologies. Benign conditions may cause elevation of CA19-9, even in the setting of previous malignancy. Persistently high CA19-9 level post-decompression raises suspicion of malignancy.

Conclusion: This case serves as a reminder for clinicians that non-specific increases in CA19-9 after curative surgery is not uncommon. We recommend careful interpretation of CA19-9 levels in combination with clinical history, physical examination and radiological investigations.

## Highlights

• CA19-9 is a valuable and widely used biomarker used in pancreatic cancer.
• Benign conditions may cause elevation of CA19-9, even in the setting of previous malignancy.
• Persistently high CA19-9 level post-decompression raises suspicion of malignancy.
• CA19-9 levels should be interpreted in combination with other investigations.

## 1 Introduction

Serum carbohydrate antigen 19-9 (CA19-9) is a valuable biomarker in pancreatic cancer, where abnormal levels often trigger the need for further confirmatory investigations. Although commonly used in diagnosis, determining resectability, and post-operative surveillance, the greatest clinical utility may be in prognostication [1]. High pre-operative CA19-9 levels confer poor post-operative prognosis.

In addition to pancreatic adenocarcinoma, the differential diagnosis for CA19-9 level elevation includes numerous non-malignant causes such as obstructive, infective and inflammatory pathology in the hepato-pancreato-biliary system [2].

This patient was managed at a quaternary hepatobiliary teaching centre. This case demonstrates that the elevation of serum CA19-9 can be non-specific and does not necessary represent oncological recurrence. Similarly, levels may be within normal limits in the setting of malignancy. Investigation for recurrence should parallel treatment of the most likely cause of CA19-9 elevation. There is a greater suspicion for an underlying recurrence in cases where CA19-9 levels do not normalise after treatment. This case report has been reported in line with the SCARE criteria [3].

## 2 Presentation of case

A previously well 78 year old male with no significant past medical or family history initially presented with an episode of acute pancreatitis that was managed expectantly. He was a non- smoker and does not consume alcohol.

Evaluation by ultrasonography (US) and computed tomography demonstrated (1) a lobulated mass in the uncinate process of the pancreas consistent with a carcinoma and (2) multiple large hepatic cysts. Endoscopic ultrasonography (EUS) showed a 4 cm solid lesion with cystic change. Further staging by positron-emission tomography scan showed a fluoro-deoxy-glucose (FDG) avid tumour in the uncinate process of pancreas with no distant metastases. Serum CA19-9 level was 22 kU/L (normal range: 0–37kU/L).

He proceeded to an uncomplicated Whipple’s procedure. Histopathology of the specimen showed R0 resection of an invasive adenocarcinoma with no vessel, neural or nodal invasion. He received adjuvant chemotherapy and did not develop any related complications.

**Fig. 1:**
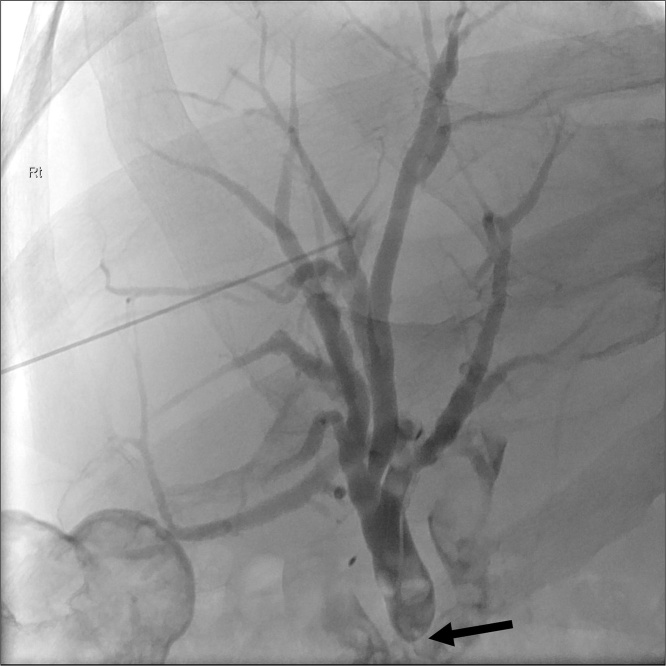
Percutaneous transhepatic cholangiography was performed and an hepatico-enterostomy anatomotic stricture was demonstrated (depicted by black arrow).

One year later, he presented with an episode of cholangitis. Restaging abdominal CT demonstrated intra-hepatic bile duct dilatation suggestive of an anastomotic stricture at the hepatico-jejunostomy with no mass lesion at the site. This was accompanied by raised serum CA19-9 level of 876kU/L. He underwent percutaneous trans-hepatic cholangiography (PTC) and cholangioplasty (Fig. 1). Biliary brushings were obtained and histopathology did not show any malignant cells. He improved clinically and his biochemical markers as well as his CA19-9 returned to normal.

**Fig. 2:**
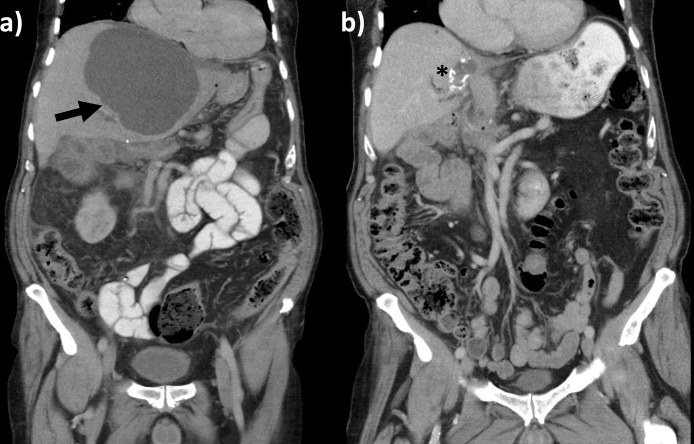
Computed tomography (CT) abdomen that was performed (a) prior to the left lateral segmentectomy and (b) 6 months after the liver surgery. Black arrow denotes a large infected hepatic cyst. Asterisk denotes the resection margin where radio-opaque stapling material can be visualised.

One month later, he developed biliary sepsis with significant biochemical and liver function derangement. Imaging confirmed evidence of infected liver cyst in the left lateral segment based on the temporal changes of cyst wall thickening and sedimentation. The largest hepatic cyst (left lateral segments) had doubled in size from 6 cm to 15 cm in diameter (Fig. 2a). Serum CA19-9 at this presentation was 297 kU/L. He was commenced on intravenous antibiotics. Ultrasound guided drainage of the hepatic cyst drained 2 L of purulent fluid. Enteric flora was cultured and biochemistry of the cyst fluid showed raised CA19-9 (>100,000UkU/L). Due to refractory sepsis, he underwent an open left lateral liver sectorectomy. Histopathology confirmed an infected biliary cystadenoma (BCA).

He was discharged eight days later with marked improvement of his biochemical investigation results and a serum CA19-9 which normalised over three months. Further imaging did not show any

radiological recurrence at six months (Fig. 2b). Six years after his curative surgery, he remained well with normalization of his biochemistry, normal serum CA19-9, and no evidence of recurrence.

## 3 Discussion

Serum carbohydrate antigen 19-9 (CA19-9) is a valuable biomarker in pancreatic cancer, where abnormal levels often trigger the need for further confirmatory investigations. Although commonly used in diagnosis, determining resectability, and post-operative surveillance, the greatest clinical utility may be in prognostication [1]. High pre-operative CA19-9 levels confer poor post-operative prognosis.

As this case demonstrates, an elevated serum CA19-9 level can result from non-malignant pathology even in the setting of prior cancer. In addition to pancreatic adenocarcinoma, the differential diagnosis for CA19-9 level elevation includes numerous non-malignant causes such as obstructive, infective and inflammatory pathology in the hepato-pancreato-biliary system.

This case report demonstrates that the elevation of serum CA19-9 can be non-specific and does not necessary represent oncological recurrence. Similarly, levels may be within normal limits in the setting of malignancy. Investigation for recurrence should parallel treatment of the most likely cause of CA19-9 elevation. There is a greater suspicion for an underlying recurrence in cases where CA19-9 levels do not normalise after treatment.

In the first occasion, cancer recurrence was excluded on clinical and radiological grounds. As seen in this case of cholangitis secondary to anastomotic stricture, CA19-9 is often elevated in benign biliary complications such as cholestasis and cholangitis [2,4]. Following treatment of the anastomotic stricture and cholangitis, CA19-9 was seen to normalise. The normalisation of CA19-9 post therapy and resolution of underlying cause is in keeping with a benign aetiology. There are a number of understood mechanisms for CA19-9 elevation in benign disease. These include bile duct cells producing increased CA19-9 with increased pressure in the biliary system, inflammatory proliferation of epithelial cells producing CA19-9, as well as intra-luminal build up for CA19-9 due to biliary obstruction. Another contributing factor is the increased permeability between biliary and sinusoidal systems causing reflux into the blood stream raising serum CA19-9 levels [1].

In the second occasion, we observed an elevation of both cystic fluid CA19-9 and serum CA19-9. Serum CA19-9 can also be elevated in simple cysts, especially in the setting of haemorrhage [5] and infection [6]. Thus, serum and cysts fluid CA19-9 cannot be readily used in the diagnosis and differentiation of benign and neoplastic cysts. It was considered that the seeding of bacteria from his episode of cholangitis or percutaneous intervention may have led to the development of an infection of his cysts one month later. The reason for raised serum CA19-9 in this setting remains unclear but is most likely related to the increased permeability of tissues during an inflammatory response. Once again, we noted that normalization of CA19-9 supports an underlying non-malignant aetiology.

CA19-9 has a role in pancreatic cancer for monitoring progress following therapy, predicting recurrence and prognostication [1,7,8]. A normal pre-operative CA19-9 level confers good prognosis. As appreciated in this case, the pre-operative CA19-9 was normal and the reported patient remains alive and disease free.

Post-operative surveillance using CA19-9 serum levels has been shown to identify recurrence up to six months prior to radiological evidence or clinical findings [9]. However, there are significant discrepancies in existing guidelines for surveillance which range from no routine surveillance to 3–6 monthly CA19-9 levels for the first 24 months in the post-operative period [10,11].

This case serves as an important reminder that benign aetiologies such as cholestasis, cholangitis, pancreatitis, hepatic cysts and abscesses may lead to elevated serum CA19-9, even in the setting of a previous malignancy. The cause of elevation is non-specific and it is often difficult to differentiate recurrences from non-malignant causes. Serum CA19-9 levels were seen to normalise post decompression of the biliary tree. If CA19-9 levels do not normalise post treatment, a suspicion of an underlying malignancy or recurrence must be raised. Careful interpretation of CA19-9 levels in combination with clinical history, physical examination and radiological investigations is highly recommended.

### 3.1 Takeaway messages

1 CA19-9 is a valuable and widely used biomarker used in pancreatic cancer.
2 Benign conditions may cause elevation of CA19-9, even in the setting of previous malignancy.
3 Investigation for recurrence should parallel treatment of the most likely cause of CA19-9 elevation.
4 If CA19-9 levels fail to normalise post decompression of biliary tree, there is increased suspicion of malignant process.
5 Serum levels should be interpreted in combination with other investigations as elevation is non-specific.

## Conflict of interest

Nil.

## Funding

Nil.

## Ethical approval

Exempt.

## Consent

Consent obtained.

## Author contribution

GG and SKG are co-first authors. GG (corresponding author) had a contribution in drafting the manuscript, editing the manuscript, finalising the manuscript and liaising with other authors. SKG had a contribution in identifying the case, planning the manuscript and drafting and editing the manuscript. CC and VM contributed to drafting the manuscript, proofing and finalising the manuscript.

## Registration of research studies

NA.

## Guarantor

Grace Gold.

Su Kah Goh.

## Provenance and peer review

Not commissioned, externally peer reviewed.
